# Effect of oxygen mass transfer rate on the production of 2,3-butanediol from glucose and agro-industrial byproducts by *Bacillus licheniformis* ATCC9789

**DOI:** 10.1186/s13068-018-1138-4

**Published:** 2018-05-23

**Authors:** Stefano Rebecchi, Davide Pinelli, Giulio Zanaroli, Fabio Fava, Dario Frascari

**Affiliations:** Department of Civil, Chemical, Environmental and Materials Engineering, University of Bologna, Via Terracini 28, 40131 Bologna, Italy

**Keywords:** 2,3-Butanediol, Oxygen transfer rate, *Bacillus licheniformis*, Process optimization, Agro-industrial by-products, Microaerobic bioproduction

## Abstract

**Background:**

2,3-Butanediol (BD) is a largely used fossil-based platform chemical. The yield and productivity of bio-based BD fermentative production must be increased and cheaper substrates need to be identified, to make bio-based BD production more competitive. As BD bioproduction occurs under microaerobic conditions, a fine tuning and control of the oxygen transfer rate (OTR) is crucial to maximize BD yield and productivity. Very few studies on BD bioproduction focused on the use of non-pathogenic microorganisms and of byproducts as substrate. The goal of this work was to optimize BD bioproduction by the non-pathogenic strain *Bacillus licheniformis* ATCC9789 by (i) identifying the ranges of volumetric and biomass-specific OTR that maximize BD yield and productivity using standard sugar and protein sources, and (ii) performing a preliminary evaluation of the variation in process performances and cost resulting from the replacement of glucose with molasses, and beef extract/peptone with chicken meat and bone meal, a byproduct of the meat production industry.

**Results:**

OTR optimization with an expensive, standard medium containing glucose, beef extract and peptone revealed that OTRs in the 7–15 mmol/L/h range lead to an optimal BD yield (0.43 ± 0.03 g/g) and productivity (0.91 ± 0.05 g/L/h). The corresponding optimal range of biomass-specific OTR was equal to 1.4–7.9 ${\text{mmol}}_{{{\text{O}}_{2} }} /{\text{g}}_{\text{CDW}} /{\text{h}}$, whereas the respiratory quotient ranged from 1.8 to 2.5. The switch to an agro-industrial byproduct-based medium containing chicken meat and bone meal and molasses led to a 50% decrease in both BD yield and productivity. A preliminary economic analysis indicated that the use of the byproduct-based medium can reduce by about 45% the BD production cost.

**Conclusions:**

A procedure for OTR optimization was developed and implemented, leading to the identification of a range of biomass-specific OTR and respiratory quotient to be used for the scale-up and control of BD bioproduction by *Bacillus licheniformis*. The switch to a byproduct-based medium led to a relevant decrease in BD production cost. Further research is needed to optimize the process of BD bioproduction from the tested byproduct-based medium.

**Electronic supplementary material:**

The online version of this article (10.1186/s13068-018-1138-4) contains supplementary material, which is available to authorized users.

## Background

2,3-Butanediol (BD) is a platform chemical whose intermediates have numerous applications, such as the production of chemicals, polymers, additives, fuels, pharmaceuticals and cosmetics [1–3]. At industrial scale, BD is produced chemically from the C4 hydrocarbon fraction of crack gases, called C4 refined II [4]. On the other hand, biotechnological BD production by fermentation of pure sugars, agricultural residues, glycerol and biomass hydrolysates represents an interesting alternative [5, 6]. Biotechnological BD production is still very limited. According to the literature data, BD bioproduction is currently performed by Global Bio-Chem Technology from corn (http://www.globalbiochem.com/html/index.php) and LanzaTech from steel mill gases [7]. An increase in bio-based BD competitiveness might be obtained through a maximization of yield and productivity, and by identifying cheaper substrates.

A large number of microorganisms are able to accumulate significant amounts of BD. The main ones belong to the genera *Klebsiella*, *Serratia*, *Enterobacter* and *Bacillus* [6]. In contrast to others, microorganisms of the genus *Bacillus* are non-pathogenic and thus more suitable for industrial applications [8–10]. In particular *Bacillus licheniformis* is known for its marked ability to produce BD [5]. As many other BD producers, this microorganism is a facultative anaerobe, capable to grow by fermentation or respiration depending on oxygen availability [11]. Under fully anaerobic conditions, the mixed-acid fermentation is active, leading to the production of acetic acid (AA), formate (FOR), lactate (LAC), BD, carbon dioxide and ethanol (EtOH) [12]. Glycerin (GLY) can also be produced, as a branch of the Embden-Meyerhof glycolytic pathway [13]. The metabolic pathway involved in BD production consists in three steps downstream glycolysis: (a) two molecules of pyruvate are converted to α-acetolactate, (b) α-acetolactate is decarboxylated to acetoin, and (c) acetoin is reduced to BD, contributing to NAD^+^ regeneration and maintenance of the intracellular redox balance [2, 14]. Under fully aerobic conditions, only O_2_ is used as electron acceptor to regenerate NAD^+^, and no acetoin reduction to BD or other fermentative processes occur. On the other hand, under oxygen-limited (or microaerobic) conditions, both fermentation and respiration take place simultaneously. Under these conditions, the production of BD and other fermentation products is strongly influenced by oxygen availability, according to the cell need to maintain the NAD^+^/NADH balance. At lower oxygen supply, glycerin and ethanol production is favored, thus diverting intermediates from the acetoin/BD route. Conversely, at increasing oxygen supply, the formation of glycerin and ethanol is limited, leading to high BD yield and selectivity. On the other hand, when oxygen supply approaches oxygen demand, the accumulation of acetoin—the oxidized precursor of BD—is favored [2, 15, 16]. Thus, a fine tuning of the oxygen transfer rate (OTR) is crucial to maintain microaerobic conditions and maximize BD yield and productivity.

Different aspects must be carefully evaluated in the development of an industrial scale microaerobic process for BD bioproduction: the identification of the optimal aeration conditions, the scale-up criteria and the control strategy. With regard to the first point, the OTR is the key parameter to maximize BD yield and productivity. As for the process *scale*-*up*, aerobic fermentations are typically scaled-up by maintaining a constant volumetric mass transfer coefficient (*k*_L_*a*) [17, 18]. On the other hand, the scale-up of microaerobic fermentations is a more complex and debated issue. In particular, while some authors recommend a constant volumetric OTR as the most effective scale-up criterion for microaerobic processes [17], others claim that due to the lack of homogeneity in large reactors other parameters should be taken into consideration for scale-up purposes [19]. A relevant parameter that has been taken into consideration in several works of BD bioproduction is the specific oxygen transfer rate per unit biomass, expressed as ${\text{mmol}}_{{{\text{O}}_{2} }} /{\text{g}}_{\text{CDW}} /{\text{h}}$ [16, 20, 21]. In microaerobic batch or fed-batch processes, as well as in continuous bio-productions with negligible O_2_ advection terms in the inlet and outlet streams, this parameter is approximately equal to the specific oxygen uptake rate ($q_{{{\text{O}}_{2} }}$). $q_{{{\text{O}}_{2} }}$ is a suitable scale-up parameter for microaerobic processes, as it takes into consideration the fact that, in reactors characterized by different microbial concentrations, the same OTR can lead to different levels of dissolved O_2_. Thus, the volumetric OTR should be controlled so as to maintain $q_{{{\text{O}}_{2} }}$ at levels between a lower threshold, below which fermentation pathways prevail, and a higher limit, above which aerobic respiration prevents BD formation. Lastly, the control of microaerobic processes has been traditionally made at constant OTR. On the other hand, some studies demonstrated that the respiratory quotient (RQ, defined as the molar ratio of carbon dioxide production rate to O_2_ uptake rate) is a more effective control parameter in BD bioproduction [20, 21].

The volumetric OTR can be varied through the *k*_L_*a*, which is in turn determined by two operating parameters: agitation and aeration in the fermenter. If a conventional mechanically agitated vessel is used, agitation is regulated by the impeller rotational speed (*N*) and aeration by the air flow rate (*Q*_G_). Thus, microaerobic conditions are the result of the combined action of *N* and *Q*_G_ on oxygen mass transfer. The identification of the OTR range that maximizes BD yield and productivity for a BD bioproduction process operated with a given strain represents a crucial step in the overall economic optimization. Furthermore, the identification of the minimum OTR that ensures the desired performances determines the minimization of the operational cost associated to mechanical agitation and air supply. A limited number of studies investigated the effect of aeration and agitation, and therefore, of *k*_L_*a*, OTR, $q_{{{\text{O}}_{2} }}$ and RQ on BD bioproduction [9, 16, 18–22]. In particular, the only two studies that attempted to identify optimal *k*_L_*a* values were based on the use of pathogenic risk group two microorganisms. In the first one, Ramachandran [23] tested the effect of different *k*_L_*a* values on BD production from lactose with a *Klebsiella oxytoca* strain, by varying the stirring rate at constant air flow rate. The optimal *k*_L_*a* resulted equal to 78 L/h, whereas lower (47 L/h) and higher (120 L/h) values led to lower yields and productivities. The second study [24] focused on BD production by *Klebsiella pneumoniae* from sucrose. The maximum BD productivity (1.5 g/h/L) was obtained at a *k*_L_*a* equal to 120 L/h, with $q_{{{\text{O}}_{2} }}$ between 3 and 6 ${\text{mmol}}_{{{\text{O}}_{2} }} /{\text{g}}_{\text{CDW}} /{\text{h}}$, whereas lower (8–55 L/h) and higher (320–620 L/h) *k*_L_*a* values led to lower productivities. Other studies [9, 25] performed a qualitative optimization of the effect of impeller rotational speed and/or air flow rate on the process performances, without any attempt to determine the corresponding *k*_L_*a* or OTR values. Of these studies, only one [9] was based on the use of a non-pathogenic strain. Other studies were aimed at identifying the OTR or $q_{{{\text{O}}_{2} }}$ range leading to optimal BD production performances [16, 19, 20, 22]. In a study of BD production from glucose by a risk group 2 strain (*Enterobacter aerogenes*), Converti et al. [16] used the final concentrations of fermentation products obtained at different $q_{{{\text{O}}_{2} }}$ values to validate the use of carbon mass and reduction degree balances for the study of microbial energetics. A thorough study was conducted on *E. aerogenes* in continuos [22], batch and fed-batch [20] bioreactors of different type and scale [19]. These studies showed that RQ is an effective control parameter, and that its optimal values range between 4 and 4.5. The best performances were obtained in the 3.5–5 ${\text{mmol}}_{{{\text{O}}_{2} }} /{\text{g}}_{\text{CDW}} /{\text{h}}$ $q_{{{\text{O}}_{2} }}$ range.

The large majority of studies on BD bioproduction by *Bacillus* species is based on the use of expensive culture media, typically containing yeast extract, beef extract or peptone along with glucose as growth substrates. Several works showed that the above-listed organic nitrogen sources lead to high BD production performances probably due to the presence of amino acids, vitamins and growth factors [9, 26, 27]. However, due to the high costs of glucose and of these nitrogen sources, the development of BD bioproduction processes based on the use of cheap byproducts or wastes as carbon and organic nitrogen sources is crucial to make BD bioproduction competitive with the petrochemical route [3, 28]. Recent studies have investigated the possibility to use sugarcane molasses [5, 29], lignocellulosic biomass and enzymatic hydrolysates of food-processing by-products [30–34] as sources of fermentable sugars for BD production with *Bacillus* strains. Conversely, only one study reported the use of a protein-rich by-product as a cheap substrate for BD production with *Bacillus* without the need to supplement the production medium with additional components [35]. An interesting substrate rich in proteins and amino acids is meat and bone meal, a product of the rendering industry. The latter converts the animal tissue (i.e., the waste of the animal slaughter industry) in value-added products, fats and a protein-rich substrate called meat and bone meal [36]. The high protein content and heating value make meat and bone meal potentially suitable for animal feeding [37, 38]. However, the emergence of bovine spongiform encephalopathy resulted in a drastic decrease of the use of this byproduct as animal feed. Thus, considering that 3.5 million tons of meat and bone meal are produced annually in Europe [37], new applications of this byproduct must be evaluated. Its high protein content makes it a potential organic nitrogen source in fermentative process [39]. To date, only two literature works report the use of meat and bone meal in biotechnological processes, aimed at the production of omega-3 polyunsaturated fatty acids [38] and cyanophycin [40].

Regarding the use of carbon sources from agro-industrial wastes for BD production, molasses represents an interesting alternative to the sugars typically utilized, due to its high content in sucrose. The use of molasses for BD bioproduction is reported in a limited number of studies, based on the use of risk group 2 pathogenic microorganisms [36, 37].

The goal of this work was to perform a preliminary optimization of the process of BD bioproduction by a non-pathogenic strain, *Bacillus licheniformis* ATCC9789. In particular, the first part of the work was aimed at identifying the volumetric OTR range that maximizes BD yield and productivity in batch tests fed with standard carbon and protein sources, so as to determine $q_{{{\text{O}}_{2} }}$ and RQ ranges to be utilized as scale-up and control parameters, respectively. The second part was aimed at performing a preliminary evaluation of the variation in process performances and costs resulting from the replacement of glucose with molasses and beef extract/peptone with chicken meat and bone meal (CMBM).

The novelties of this work are:

- The study of the aeration conditions for BD production by a non-pathogenic strain, whereas all the studies that included this aspect in a quantitative way are based on the use of pathogenic risk group 2 strains;
- The identification of optimal $q_{{{\text{O}}_{2} }}$ and RQ ranges to be used for scale-up and control purposes for BD production by a non-pathogenic strain, whereas the previous studies followed a semi-qualitative approach mainly based on *k*_*L*_*a* or aeration conditions;
- The replacement of the expensive protein and C sources typically used in the previous studies with meat and bone meal, an extremely cheap byproduct of the meat production industry never tested before for BD bioproduction, in combination with molasses, a low-value byproduct of the sugar production industry.

## Methods

### Evaluation of oxygen mass transfer rate (OTR) in microaerobic conditions

The OTR of a batch bioprocess can be evaluated by means of two approaches, that derive respectively from the liquid-phase and gas-phase oxygen mass balances written under the assumption of perfectly mixed fluid dynamic model:

1 $$V_{\text{L}} \cdot \frac{{{\text{d}}c_{\text{L}} }}{{{\text{d}}t}} = {\text{OTR}} \cdot V_{\text{L}} - {\text{OUR}} \cdot V_{\text{L}}$$

2 $$V_{\text{G}} \cdot \frac{{{\text{d}}C_{\text{G}} }}{{{\text{d}}t}} = Q_{\text{G}}^{\text{IN}} \cdot C_{\text{G}}^{\text{IN}} - Q_{\text{G}}^{\text{OUT}} \cdot C_{\text{G}}^{\text{OUT}} - {\text{OTR}} \cdot V_{\text{L}}$$

where $C_{\text{L}}$ and $C_{\text{G}}$ are the oxygen concentrations in the liquid and gas phase, $Q_{\text{G}}^{\text{IN}}$ and $Q_{\text{G}}^{\text{OUT}}$ the entering and exiting gas volumetric flow rates, OUR the oxygen uptake rate in the liquid phase and OTR the gas–liquid oxygen transfer rate. The latter can be calculated as:

3 $${\text{OTR}} = k_{\text{L}} a \cdot \left( {\frac{{C_{\text{G}} }}{{m_{{\text{O}}_{2}} }} - C_{\text{L}} } \right)$$

where $k_{\text{L}} a$ is the oxygen volumetric mass transfer coefficient, $m_{{{\text{O}}_{2} }}$ the gas/liquid partition coefficient for oxygen and $C_{\text{G}}$ the local value of O_2_ concentration in the gas phase. In this work, the gas phase was considered perfectly mixed. The measured $C_{\text{G}}^{\text{OUT}}$ was, therefore, assumed to apply in the entire gas phase. The variations between $C_{\text{G}}^{\text{IN}}$ and $C_{\text{G}}^{\text{OUT}}$ were, in anyway, rather small (in average, $C_{\text{G}}^{\text{OUT}} = 0.8C_{\text{G}}^{\text{IN}}$).

In the microaerobic conditions required for an optimal BD production, $C_{\text{L}}$ can be assumed equal to zero with a negligible error, and therefore, ${\text{d}}C_{\text{L}} /{\text{d}}t$ is also null. Thus, Eqs. (1–3) become:

4 $${\text{OTR}} = {\text{OUR}} = k_{\text{L}} a \cdot \frac{{C_{\text{G}} }}{{m_{{\text{O}}_{2}} }}$$

5 $${\text{OTR}} = \left( {Q_{\text{G}}^{\text{IN}} \cdot C_{\text{G}}^{\text{IN}} - Q_{\text{G}}^{\text{OUT}} \cdot C_{\text{G}}^{\text{OUT}} } \right)/V_{\text{L}}$$

Hence, the OUR equals the OTR and the latter, in the studied microaerobic conditions, determines the actual bioprocess rate. Therefore, a very precise tuning of OTR is crucial to maintain the required microaerobic conditions. Finally, the biomass-specific OUR (or $q_{{{\text{O}}_{2} }}$) can be calculated dividing OTR by the biomass concentration, expressed in this work as cell dry weight. As in a batch process biomass concentration increases with time, $q_{{{\text{O}}_{2} }}$ decreases during the fermentation, if a constant OTR is applied.

Equations (4) and (5), therefore, represent two alternatives for evaluating the OTR for each tested experimental condition. Preliminary calculations indicated that even if the first approach—OTR evaluation from Eq. (4)—was affected by a lower 95% confidence interval than the second one—OTR evaluation from the gas-phase oxygen mass balance Eq. (5)—the difference is limited: 13% versus 16%, in relative terms. The OTR evaluated for each experimental condition with the second approach was, therefore, utilized in this work as it has the advantage of a more straightforward determination, not affected by the fluid dynamic conditions in the bioreactor. On the other hand, the *k*_L_*a*-based assessment of OTR Eq. (4) was used to validate the first approach and to make an approximate prediction of the actual OTR before running each experiment. The experimental technique used to assess the *k*_L_*a* of each test is illustrated as detail in Additional file 1: Table S1 [41, 42].

### Microorganism and inoculum preparation

*Bacillus licheniformis* ATCC 9789 was obtained from American Type Culture Collection. The strain was stored at − 80 °C in glycerol stock. The medium used for the seed culture growth was composed by 5 g/L beef extract and 3 g/L peptone from soybean. The seed culture was prepared by inoculating 0.5 mL of stock culture into 250 mL shake flasks each containing 50 mL of medium. The flasks were incubated at 30 °C and 150 rpm for 24 h.

### BD production tests

Fermentations were carried out in a BIOSTAT B-Twin bioreactor (Sartorius AG, Germany) equipped with pH, temperature, foam and dissolved oxygen control and filled with 1 L of fermentation medium. The bioreactor is characterized by a 0.230 m total height, 0.081 m liquid height and 0.130 m internal diameter. Agitation was transmitted to the liquid by a single six-blade Rushton turbine (0.05 m diameter). Air was introduced through a perforated ring located under the turbine.

BD bioproduction tests were carried out at 30 °C and pH 6. After medium sterilization (121 °C, 20 min), the liquid phase was saturated with air and the seed culture was inoculated into the fermenter (5% v/v). The specific operational conditions of the BD production tests are reported in the left-hand part of Table 1. Tests SM-1 to SM-V7 (Table 1) were conducted with a standard medium (SM) optimized for BD bioproduction having the following composition: beef extract 10 g/L, peptone 10 g/L, NaCl 5 g/L, glucose 40 g/L [43]. In tests BP-1 to BP-4 (Table 1) the expensive components of the standard medium were replaced with cheap byproducts (BP), as illustrated in detail below.

**Table 1:** Overview of the BD production tests: experimental conditions, oxygen mass transfer coefficient, oxygen transfer rates, liquid phase concentrations

Test ID	Type of medium	Test type	*N* (rpm)	*Q*_G_ (L/min)	*k*_L_*a* (1/h)^a^	OTR (mmol/L/h)^b^	$q_{{{\text{O}}_{2} }}$ (mmol/g_CDW_/h)^c^	RQ (–)^d^	CO_2, L_ (mg/L)^e^	BD (g/L)^f^	AC (g/L)^f^	GLY (g/L)^f^	Ethanol (g/L)^f^	CDW (g/L)^g^
SM-1	Standard	Preliminary test	Variable	0.31	Variable	Variable	Variable		0.008	8.3	11.7	0.0	0.0	3.6–6.3
SM-2	Standard	CCCD	250	0.10	6.5 ± 2.2	1.6 ± 0.5	0.7–0.4	7.0	< 0.008	17.6	0.0	9.4	0.1	2.3–3.7
SM-3	Standard	CCCD	198	0.31	8.7 ± 0.8	1.7 ± 0.2	0.7–0.5	4.0	< 0.008	15.6	0.0	9.5	1.0	2.4–3.2
SM-4	Standard	CCCD	250	0.51	13.0 ± 2.4	3.3 ± 0.5	1.2–0.8	1.8	< 0.008	17.0	0.1	7.6	0.8	2.7–4.2
SM-5	Standard	CCCD	375	0.05	13.6 ± 1.5	1.0 ± 0.2	0.4–0.2	5.7	< 0.008	19.7	0.2	6.8	1.2	2.6–4.6
SM-6	Standard	CCCD	375	0.31	21.8 ± 2.4	1.6 ± 0.2	0.6–0.3	14.1	< 0.008	18.0	0.1	5.8	0.4	2.8–5.2
SM-7	Standard	CCCD	375	0.59	32.2 ± 3.3	7.3 ± 0.7	2.3–1.4	2.1	< 0.008	17.1	0.8	3.9	0.7	3.2–5.2
SM-8	Standard	CCCD	500	0.10	46.6 ± 3.9	10.9 ± 0.8	2.6–1.7	2.5	< 0.008	18.4	1.7	0.3	0.0	4.2–6.4
SM-9	Standard	CCCD	500	0.51	80.8 ± 8.5	17.4 ± 1.8	3.6–2.6	1.9	< 0.008	16.3	2.4	0.1	0.0	4.8–6.8
SM-10	Standard	CCCD	552	0.31	84.7 ± 5.8	22.7 ± 1.2	8.4–5.2	1.1	3.1	2.0	11.0	0.0	0.0	2.7–4.3
SM-V1	Standard	Validation test	460	0.10	34.4 ± 2.9	9.4 ± 0.7	4.3–1.6	2.1	< 0.008	19.7	2.1	1.2	0.0	2.2–5.7
SM-V2	Standard	Validation test	430	0.24	26.1 ± 4.8	3.5 ± 1.1	1.5–0.6	3.0	< 0.008	16.8	1.4	0.4	0.0	2.4–5.7
SM-V3	Standard	Validation test	375	0.14	20.3 ± 0.3	1.2 ± 0.1	0.5–0.2	7.6	< 0.008	17.5	0.0	5.5	0.7	2.7–5.1
SM-V4	Standard	Validation test	405	0.51	39.4 ± 2.3	14.1 ± 0.9	5.0–2.4	1.9	< 0.008	19.3	3.8	0.0	0.0	2.8–5.8
SM-V5	Standard	Validation test	425	0.43	38.5 ± 2.5	15.1 ± 1.1	7.9–2.7	1.8	< 0.008	19.0	2.7	0.0	0.0	1.9–5.5
SM-V6	Standard	Validation test	478	0.51	72.0 ± 7.2	20.4 ± 1.2	9.8–4.1	1.8	< 0.008	7.3	10.5	0	0	2.1–5.0
SM-V7	Standard	Validation test	800	0.81	142 ± 15	28.7 ± 1.1	6.1–4.5	^h^	5.2	3.2	9.9	0.0	0.0	4.7–6.4
BP-1	Glucose + CMBM^i^	Byproduct test	405	0.50	22.0 ± 0.1	6.1 ± 0.7	1.4–0.7	^h^	< 0.008	16.9	5.2	0.9	0	4.3–8.6
BP-2	Molasses + CMBM^i^	Byproduct test	405	0.50	20.9 ± 0.2	5.8 ± 0.7	1.2–0.8	^h^	< 0.008	11.5	2	0.5	0	4.9–6.9
BP-3	Molasses + CMBM^i^	Byproduct test	460	0.10	29.5 ± 0.5	8.2 ± 1.0	1.7–1.2	^h^	< 0.008	9.4	0.9	0	0	4.9–6.6
BP-4	Molasses + CMBM^i^	Byproduct test	500	0.10	47.5 ± 3.6	13.1 ± 1.6	3.1–1.6	^h^	< 0.008	10.1	3.7	0	0	4.2–8.2

^a^Evaluated experimentally through the dynamic method. ^b^ Volumetric OTR, evaluated according to Eq. (5). ^c^ O_2_ specific utilization rate (or biomass-specific OTR) evaluated at the onset and at the end of the actual phase of BD production, respectively. ^d^ Respiratory quotient, evaluated as molar ratio of carbon dioxide production rate to O_2_ uptake rate. ^e^ Average O_2_ concentration in the liquid phase during the BD production phase. ^f^ Liquid phase concentrations at the end of the BD production phase. ^g^ Cell dry weight concentration evaluated at the onset and at the end of the actual phase of BD production, respectively. ^h^ Not available. ^i^ Chicken meat and bone meal

**Fig. 1:**
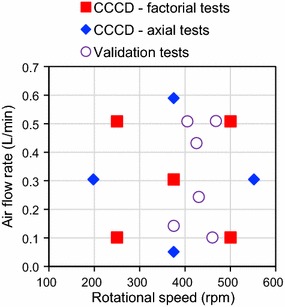
Aeration conditions utilized in the CCCD and validation standard medium tests

The first standard medium test (SM-1) aimed at verifying preliminarily the possibility to use the dissolved oxygen concentration automatic control system to guarantee the optimal microaerobic condition required for BD bioproduction. The dissolved O_2_ concentration was set to the quantification limit of the oxygen probe, i.e., 0.008 mg/L. All other tests were carried out at constant agitation (*N*) and aeration (*Q*_G_) during the whole process (i.e., during both the cell growth and BD production phases). Different operational conditions in terms of *N* and *Q*_G_, and therefore, *k*_L_*a* and OTR, were tested to identify the OTR range leading to the optimal BD production performances with the standard medium (SM-2 to SM-V7; Table 1). In particular, in a first set of experiments (CCCD tests, SM-2 to SM-10) *N* and *Q*_G_ values were selected according to a design of experiment methodology based on the Central Composite Circumscribed Design (CCCD) articulated in two levels (+ 1, − 1) for each factor (*N* and *Q*_G_) (2^2^ full factorial design) and a central point, that was repeated three times, augmented with four axial points (*α* = 1.41). The lower and higher levels for the factorial points were 0.1 and 0.5 L/min for *Q*_G_ and 250 and 500 rpm for *N*. These levels were selected so as to locate all points—including the axial ones—within overall *N* and *Q*_G_ ranges (200–550 rpm and 0.05–0.6 L/min, respectively) identified as fluid-dynamically correct according to a preliminary visual analysis carried out in the absence of inoculum (i.e., absence of agitator flooding, due to too high *Q*_G_ and too low *N*, or of a pronounced central vortex with substantial surface aeration, due to too high *N*). The resulting 9 CCCD conditions are represented in Fig. 1 (squares and diamonds). A second group of tests (validation tests, SM-V1–SM-V7) included 6 OTR values (SM-V1–SM-V6) falling within the optimal range identified in the CCCD tests (empty circles in Fig. 1, see the "Results and discussion" section for more details), and an additional fully aerobic test (SM-V7, *N* = 800 rpm, *Q*_*G*_ = 0.81 L/min). Test SM-V5 was repeated three times.

In the tests conducted with cheap byproducts as substrate (BP tests), chicken meat and bone meal was used as alternative source of proteins and molasses as alternative source of sugars. The tested chicken meat and bone meal had the following composition: proteins 65.7%, fats 14.1%, carbohydrates 4.5%, ashes 15.5%, humidity 0.7%. The tested molasses had the following composition: glucose 30%, fructose 24%, ashes 7%, humidity 13%. In particular, in test BP-1, only peptone and beef extract were replaced with chicken meat and bone meal (30 g/L, so as to maintain the total protein concentration of the standard medium tests, equal to 19.7 g_protein_/L). In tests BP-2 to BP-4, in addition to replacing peptone and beef extract with chicken meat and bone meal, glucose was replaced with molasses (74 g/L, so as to maintain the total sugar concentration of the standard medium tests, equal to 40 g/L). The aeration conditions (*N* and *Q*_G_) of the BP tests (Table 1) were selected on the basis of the optimal values obtained in the tests with standard medium (see “Results and discussion” for more details).

### Indicators of BD production performances

Three main indicators were selected to characterize the BD production performances in each test: overall BD yield ($Y_{\text{BD}}$), calculated as (final BD mass)/(initial glucose mass); average BD productivity ($P_{\text{BD}}$), calculated as (final BD concentration)/(total fermentation time, including the aerobic cell growth phase); and maximum BD production rate ($r_{\text{BD}}$), calculated as the average slope of the plot of BD concentration versus time during the microaerobic phase. The latter indicator, therefore, represents the upper limit to which—under each experimental condition—the average BD productivity tends if the duration of the aerobic cell growth phase is negligible.

Three more indicators were utilized to provide more information on the outcome of each test: the acetoin ($Y_{\text{AC}}$) and glycerol ($Y_{\text{GLY}}$) overall yields, calculated as (final acetoin or glycerol mass)/(initial glucose mass), and the glucose consumption rate during the BD production phase ($r_{\text{GL}}$), calculated as the average slope of the plot of glucose concentration versus time, limitedly to the microaerobic phase.

The tests conducted in triplicate (SM-6 and SM-V5) were used to estimate an average uncertainty (expressed as 95% confidence interval) relative to each indicator of BD production performance.

### Analytical methods

Glucose, BD, acetoin, glycerol and ethanol were analyzed with an Agilent 1260 Series HPLC equipped with an Agilent Hi-Plex H column (8 μm, 300 × 7.7 mm) and a refractive index detector (RID) under the following conditions: mobile phase H_2_SO_4_5 mM, flow rate 0.6 mL/min, injection volume 0.5 μL, column temperature 65 °C [5]. Oxygen concentration in the gas phase was measured with an Agilent 3000 MicroGC coupled with a TCD detector (injector temperature 90 °C; column temperature 60 °C; sampling time 20 s; injection time 50 ms; column pressure 25 psi; run time 44 s; carrier gas N_2_). Biomass Growth was measured by optical density (OD) at 600 nm using a Prixma spectrophotometer (Fulltech Instruments). Optical density was converted to cell dry weight (CDW) using a proportionality factor (0.385) obtained from the OD vs CDW calibration curve. The calibration curve was obtained by measuring OD and CDW in samples collected at different times during a fermentation. These samples were centrifuged (8000 rpm, 10 min) and the cells were washed with de-ionized water. The cell dry weight was measured after drying cells overnight at 105 °C. Proteins were measured with the Bradford method [44], using the commercial protein assay dye reagent provided by BioRad (Milano, Italy).

## Results and discussion

### Comparison between experimental and *k*_L_*a*-based OTR values

As illustrated in “Methods”, the OTR relative to each test was evaluated by means of the O_2_ mass balance in the gas phase Eq. (5). As the correct evaluation of OTR represents a crucial element of this work, the alternative *k*_L_*a*-based evaluation Eq. (4) was used to verify that the experimental OTR values are consistent with the mass transfer properties measured before the fermentation. To this purpose, the *k*_L_*a* associated to each aeration condition and to each medium type was measured experimentally according to a variation of the dynamic method described in Additional file 1: Table S1. To validate the experimentally determined coefficients, the *k*_L_*a* values were also estimated by means of two correlations widely used in the literature for a stirred tank reactor [41, 45, 46], as illustrated in Additional file 1: Table S2. The average deviations between experimental and theoretical *k*_L_*a* values resulted equal to 23% for the first correlation [41] and 37% for the second [45], indicating that the experimental *k*_L_*a* measurements are acceptably reliable. The observed deviations can be ascribed to the different geometric ratios between the standard vessel used to obtain the literature correlations and the actual vessel used in this work. The measured *k*_L_*a* values were then used to calculate the *k*_L_*a*-based OTR associated to each fermentation. The experimental and *k*_L_*a*-based OTR values resulted acceptably well correlated (*R*^2^ = 0.87; Additional file 1: Fig. S1). However, a systematic deviation equal as an average to 25% was observed between the two sets of OTR values. The latter could be ascribed to the change in fermentation medium properties during the experiments.

### BD production performances in the standard medium CCCD tests

The preliminary test (SM-1, Table 1) was aimed at verifying the possibility to use the dissolved oxygen concentration automatic control system of the fermenter to grantee the optimal microaerobic condition required for BD bioproduction. In this test, after an initial phase of cell growth conducted under full aerobic conditions, the air flow rate (*Q*_G_) was set to 0.31 L/min (0.31 vvm), whereas the impeller rotational speed was used by the O_2_ concentration control system to maintain a dissolved O_2_ concentration equal to the quantification limit of the oxygen probe, i.e., 0.008 mg/L. The O_2_ control system effectively maintained the assigned dissolved O_2_ concentration, but the process performances were poor: the low overall BD yield (0.20 g_BD_/g_GL_) and average productivity (0.38 g/h/L) were associated to a high acetoin yield (0.28 g_AC_/g_GL_). This outcome indicates that the imposed O_2_ concentration (0.008 mg/L) was higher than the microaerobic conditions required for an optimal BD production by *Bacillus licheniformis* ATCC 9789.

**Fig. 2:**
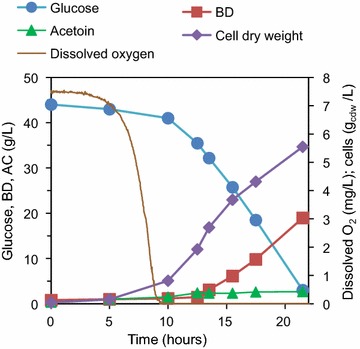
Profiles of substrate, products, cells and dissolved O_2_ concentrations versus time in a typical fermentation conducted with the standard medium (test SM-V5)

Given the unsatisfactory results of test SM-1, all the subsequent tests were conducted without automatic O_2_ control, at fixed values of *N* and *Q*_G_, following the procedure illustrated in the Materials and methods. Thus, starting from fully aerobic conditions, as the cells grew the OUR increased. As a result, the dissolved oxygen gradually decreased until—after 4 to 15 h, depending on the operational conditions—microaerobic conditions were reached. Typical profiles of substrate, products, cells and dissolved O_2_ concentration versus time obtained with this procedure are shown in Fig. 2.

**Fig. 3:**
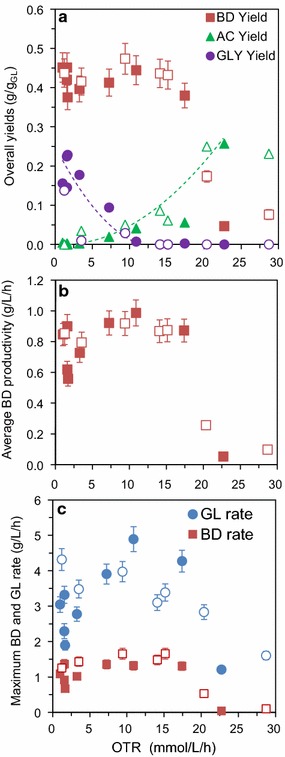
Standard medium tests: performance indicators versus OTR. Full symbols refer to the CCCD tests (SM-2 to SM-10), whereas empty symbols refer to the validation tests (SM-V1 to SM-V7). The curves reported in some subplots represent just interpolating lines introduced to increase the figure readability. These curves do not represent model simulations. BD, AC or GLY overall yield = (final BD, AC or GLY mass)/(initial glucose mass); average BD productivity = (final BD concentration)/(total fermentation time, including the aerobic cell growth phase); maximum BD production rate = average slope of the plot of BD concentration versus time during the microaerobic phase; maximum GL consumption rate = average slope of the plot of GL concentration versus time during the microaerobic phase

To identify the optimal aeration conditions for BD production, a set of tests was carried out at different *N* and *Q*_G_, which were initially selected using a Central Composite Circumscribed Design (CCCD). The design of experiment (DoE) approach is a widely utilized tool for the optimal selection of the experimental condition to be tested and to address process optimization. In the field of BD bioproduction, the DoE has been previously applied for optimizing the culture medium [26, 47, 48] and the process temperature and pH [49, 50], but never for optimizing the aeration conditions. The OTR values estimated for each test according to Eq. (5) are reported in Table 1, together with the final concentrations of BD, acetoin, glycerol, ethanol and cells. The plots of the six selected performance parameters versus OTR are represented in Fig. 3 with full symbols for the CCCD tests (SM-2–SM-10) and empty symbols for the validation tests (SM-V1–SM-V7).

With the exception of tests SM-10, that reached fully aerobic conditions during the BD production phase (Table 1), in all CCCD tests the plots of glucose, BD, cells and—where present—acetoin concentration versus time were about linear and not exponential. This finding indicates that the biological activity was controlled by the oxygen mass transfer rate, which—according to Eq. (4)—remained constant during each BD production phase. This represents an indication that microaerobic conditions were actually achieved. In agreement with this observation, these tests provided satisfactory values of both average BD productivity (0.56–0.98 g/L/h) and overall BD/glucose yield (0.38–0.47 g/g), corresponding to 75–94% of the theoretical maximum yield (0.50 g/g), i.e., the yield that could be obtained if BD was the only product and no biomass formation from glucose occurred. In line with the rather high BD yields, rather low acetoin and glycerol yields and negligible ethanol concentrations were found in these tests. Conversely, the performances of the aerobic tests were poor (*Y*_BD_ = 0.05–0.08 g/g, *P*_BD_ = 0.05–0.20 g/L/h).

The overall BD yield relative to the CCCD tests (full symbols in Fig. 3a) presents a roughly constant value (0.42 ± 0.03 g/g) in the 1–11 mmol/L/h OTR range, followed by a rapid decrease in the 17–23 mmol/L/h range, due to the shift to aerobic conditions. Indeed at high OTR, the most abundant product is acetoin, and O_2_ is mainly used instead of acetoin to maintain the redox balance. 1–11 mmol/L/h was thus identified as the optimal OTR range that leads to high yields of BD production by *Bacillus licheniformis* ATCC9789 in the standard medium tests. Within this OTR range, the glycerol yield gradually decreased and reached zero in correspondence with an OTR equal to 11 mmol/L/h, whereas the acetoin yield slowly increased with increasing OTR, with a maximum value of 0.04 g/g.

In terms of average BD productivity (Fig. 3b, CCCD tests indicated with full symbols), a rapid increase up to an OTR equal to 3 mmol/L/h—corresponding to the OTR interval, where the BD production process was completely controlled by the oxygen transfer rate—was followed by a roughly constant value (0.88 ± 0.08 g_BD_/L/h) in the 3–17 mmol/L/h OTR range. Similarly, the maximum BD production rate (*r*_BD_, Fig. 3c, CCCD tests indicated with full symbols) showed an increase, followed by a roughly constant value (1.25 ± 0.07 g_BD_/h/L) for OTR values in the 7–17 mmol/L/h range. At OTR > 17 mmol/L/h, the shift to aerobic conditions determined a drastic decrease in average BD productivity and maximum BD production rate. $r_{\text{BD}}$, evaluated as the average rate relative to the BD production phase, represents the maximum value to which the average BD productivity could tend if the duration of the cell production phase becomes negligible. 7–17 mmol/L/h was thus identified as the OTR range leading to optimal values of average BD productivity and maximum production rate. These trends confirm that BD production by *Bacillus licheniformis* ATCC9789 is controlled by the O_2_ transfer rate up to an OTR of about 3 mmol/L/h, whereas above this threshold the shift to a process controlled by the biological kinetic leads to a process rate almost independent of the O_2_ transfer rate.

In the perspective to identify an optimal OTR range for BD production by *Bacillus licheniformis* ATCC9789 in the tested experimental conditions, the optimal ranges identified for the single performance indexes on the basis of the data reported in Fig. 3 are, therefore, 1–11 mmol/L/h for the overall BD yield, and 7–17 mmol/L/h for the maximum BD rate and average productivity. Only a comprehensive economical evaluation could lead to the identification of the optimal OTR value, i.e., of the best compromise between the conditions that favor either yield or production rate/productivity. As the economic analysis of the process is beyond the scope of this work, the conclusion that can be drawn from the above-illustrated analysis is that the optimal OTR value lies in the region of overlap between the ranges that optimize the BD yield and production/productivity, i.e., in the 7–11 mmol/L/h range. The drastic drop in BD production performances observed for OTR > 17 mmol/L/h indicates that a careful OTR control is crucial to optimize the BD production process by *Bacillus licheniformis* ATCC9789, and that the OTR set point should be controlled to a value sufficiently lower than the borderline value of 17 mmol/L/h.

The evaluation of the optimal OTR was integrated by a direct assessment of the best operating conditions in terms of impeller rotational speed *N* and air flow rate *Q*_G_, based on the response surface methodology (RSM). The RSM, based on the data relative to the CCCD tests (SM-2 to SM-10, Table 1), was applied to the overall BD yield and average productivity, considered the two most relevant performance parameters. As reported in detail in Additional file 1: Table S3 and Fig. S2, the RSM-based optimal aeration condition obtained by combining the best-fitting polynomial expressions relative to BD yield and productivity was *N *= 462 rpm and *Q*_G_ = 0.1 L/min [51]. The corresponding OTR, equal to 8.5 mmolO_2_/L/h, is in good agreement with the optimal OTR range obtained from the CCCD tests (7–11 mmolO_2_/L/h).

### BD production performances in the standard medium validation tests

To provide a confirmation of the BD production performances obtained in the OTR range identified as optimal on the basis of the CCCD tests (7–11 mmol/h/L) and to add an experimental point in the OTR range where the metabolic regime turns from microaerobic to aerobic, seven validation tests (SM-V1 to SM-V7) were conducted with the same standard medium. The aeration conditions of these tests, reported in Table 1 and in Fig. 1, were designed so as to cover an OTR range (3–15 mmol/L/h) slightly wider than the one previously identified as optimal. To this purpose, the OTR values predicted on the basis of *k*_L_*a* were used. It should be noted that, on the basis of Eqs. (4) and (5), the actual OTR of each test depends on *k*_L_*a*, that can be predicted from the aeration conditions, and on the average O_2_ concentration in the gas phase, that cannot be predicted a priori in a precise way. Therefore, the actual OTR can become different than the desired one.

The performances of the validation tests, reported with empty symbols in Fig. 3, were in good agreement with those of the CCCD tests, with an average 0.44 ± 0.02 g/g BD yield (4% increase), 0.86 ± 0.04 productivity (2% decrease) and 1.50 ± 0.15 production rate (20% increase). However, it should be noted that the maximum glucose consumption rate of the validation tests was characterized by a decreasing trend, whereas the corresponding rate of the CCCD test presented an increasing trend. The results of the validation tests corroborate the identification of the 7–11 mmol/L/h interval as a suitable OTR range and suggest that a higher upper limit, equal to 15 mmol/L/h, can be used. Therefore, 7–15 mmol/L/h can be considered the final optimal range for BD production by *Bacillus licheniformis* ATCC9789 with the standard medium. Combining the results relative to the CCCD and validation tests, the average performances obtained in the optimal OTR interval are: average BD yield 0.44 ± 0.2 g/g; average BD productivity 0.91 ± 0.05 g/L/h; maximum BD production rate 1.49 ± 0.15 g/L/h.

Finally a further test (SM-V7) was run to confirm the drastic drop in the BD production performances by the tested strain under fully aerobic conditions. In this test, an 800 rpm rotational speed and an 0.81 L/min gas flow rate led to a 29 mmol/L/h OTR, and to an average oxygen concentration equal to 5 mg/L. The BD production performances were extremely poor (*Y*_BD_ = 0.05 g/g, BD productivity = 0.05 g/h/L), in agreement with those of the other aerobic test, SM-10.

The overall BD yield obtained with *Bacillus licheniformis* ATCC9789 in this study is in good agreement with those reported by other studies conducted both with the same microorganism (0.42–0.47 g/g [10, 26, 43, 52]) and with pathogenic bacteria (0.41–0.49 g/g [20, 23, 53–55]). Conversely, the range of average BD productivity reported in the literature is quite large (0.1–6 g/L/h), due to the influence of several variables, such as cell concentration and operating mode (batch versus continuous), on this parameter. Several studies report that the switch from wild type strains to genetically modified ones determined a significant increase in BD productivity [56–60]. As for the mass transfer coefficient *k*_L_*a*, the optimal range (32–47 L/h; Table 1) obtained in this work is lower than the best *k*_L_*a* values previously identified for risk group 2 pathogen strains (80–120 L/h; [23, 24]).

While the volumetric OTR is a crucial parameter to optimize the performances of microaerobic processes such as BD bioproduction, a relevant parameter to be taken into consideration for the process scale-up is the specific oxygen transfer rate per unit biomass, approximately equal for microaerobic processes to the specific oxygen uptake rate $q_{{{\text{O}}_{2} }}$. The data collected in this work in the optimal range of volumetric OTR were thus used to assess a corresponding $q_{{{\text{O}}_{2} }}$ range to be used as a scale-up criterion for BD production by *Bacillus licheniformis* ATCC9789. It should be noted that, in batch conditions, the cell concentration increases with time and that—even if all the tests started at the same initial cell and glucose concentration, the final cell concentrations increased with increasing OTR (Table 1). Thus, as the OTR was maintained constant during each test, $q_{{{\text{O}}_{2} }}$ decreased with time. The $q_{{{\text{O}}_{2} }}$ values at the beginning and at the end of the BD production phase of each test were calculated by dividing the constant OTR by the cell concentrations measured at the same instants or at the nearest sampling times (Table 1). The beginning of BD production was typically observed 2–3 h after the attainment of the microaerobic condition. As shown in Table 1, the $q_{{{\text{O}}_{2} }}$ values corresponding to the identified optimal OTR values ranged from 1.4 to 7.9 mmol/g_CDW_/h. $q_{{{\text{O}}_{2} }}$ values < 1 mmol/g_CDW_/h, corresponding to anaerobic conditions, led to a considerable production of GLY (SM 2–6), whereas initial $q_{{{\text{O}}_{2} }}$ values > 8 mmol/g_CDW_/h led to a significant AC co-production (SM-10, SV-5, SV-6). The $q_{{{\text{O}}_{2} }}$ range obtained in this work is in agreement with those reported in previous studies conducted with pathogenic strains: 3–6 mmol/g_CDW_/h for a sucrose-fed batch BD bioproduction by *Klebsiella pneumoniae* [24]; 0.8–2.5 mmol/g_CDW_/h for a batch process conducted with *E. Aerogenes* and fed with glucose [16]; 3.6–3.7 mmol/g_CDW_/h for a continuous process [22] and 4–5 mmol/g_CDW_/h for a batch process, using *E. Aerogenes* [20] and glucose.

Lastly, a useful parameter for the control of microaerobic processes is the respiratory quotient RQ. As shown in Table 1, the RQ values corresponding to the optimal OTR range varied from 1.8 to 2.5. Although the RQ estimates are characterized by a relatively high 95% confidence interval (equal to about 30%), 1.8–2.5 can be identified as the optimal RQ range for BD production by *Bacillus licheniformis* ATCC9789 in the standard medium tests. The optimal RQ values identified in this work are significantly lower than the best values reported by Zeng [20] for a glucose-fed BD bioproduction conducted with *E. aerogenes* (4–4.5). This difference can be ascribed not only to the different strain used in the two studies but also to the different media used (glucose + protein sources in this study, only glucose in Zeng’s work [20]) and to the different performance indicators selected. Overall, the analysis of the literature on BD bioproduction indicates that the process performances and the optimal OTR, $q_{{{\text{O}}_{2} }}$ and RQ values depend on the type of bacterial strain utilized.

### Preliminary evaluation of the BD production performances in the tests fed with agricultural byproducts

The second part of the work was aimed at performing a preliminary evaluation of the change in BD production performances associated to the progressive replacement of the expensive medium components used in the standard medium tests (glucose, beef extract, peptone) with cheap sources of sugars (molasses) and proteins (chicken meat and bone meal). The OTR, $q_{{{\text{O}}_{2} }}$ and BD production performance parameters obtained in these tests are compared in Table 2 with the corresponding average values obtained in the standard medium tests with OTR values in the optimal range (7–15 mmol/h/L).

**Table 2:** Oxygen transfer rate and BD production performances obtained in the agricultural byproduct tests with 95% confidence intervals, and corresponding average values obtained in the standard medium tests with OTR values in the 6–8 mmol/h/L range

Test ID	Type of medium	OTR (mmol L/h)	$q_{{{\text{O}}_{2} }}$ (mmol/g_CDW_/h)	*Y*_BD_ (g_BD_/g_sugar_)	*P*_BD_ (g/L/h)	*r*_BD_ (g/L/h)
BP-1	Glucose + CMBM^a^	6.1 ± 0.7	1.4–0.7	0.38 ± 0.03	0.35 ± 0.03	0.58 ± 0.05
BP-2	Molasses + CMBM^a^	5.8 ± 0.7	1.2–0.8	0.22 ± 0.02	0.54 ± 0.05	1.38 ± 0.13
BP-3	Molasses + CMBM^a^	8.2 ± 1.0	1.7–1.2	0.25 ± 0.02	0.42 ± 0.04	1.07 ± 0.10
BP-4	Molasses + CMBM^a^	13.1 ± 1.6	3.1–1.6	0.20 ± 0.02	0.43 ± 0.04	1.10 ± 0.10
Standard medium tests in the optimal range^b^	Standard	7–15	1.4–7.9	0.44 ± 0.02^b^	0.91 ± 0.05^b^	1.49 ± 0.15^b^

^a^Chicken meat and bone meal

^b^Average value obtained in the standard medium tests characterized by an OTR in the 7–15 mmol/h/L range, with 95% confidence interval

The first two by-product tests (BP-1 and BP-2) were conducted under the aeration conditions (*N* = 405 rpm, *Q*_G_ = 0.51 L/min) that had led in the standard medium tests to optimal BD production performances and to OTR and $q_{{{\text{O}}_{2} }}$ values within the optimal ranges (14.1 mmol/L/h and 2.4–5 mmol/g_CDW_/h, respectively). In test BP-1, where beef extract and peptone were replaced with CMBM but glucose was maintained, the BD yield was slightly lower than that obtained in the optimal SM tests (− 14%), whereas a 60% decrease was observed for the BD average productivity and maximum production rate. In BP-2, the additional replacement of glucose with molasses determined a less marked decrease in BD average productivity and maximum production rate (7–41%, in comparison with the optimal SM tests), whereas the BD yield dropped significantly (50% decrease). Both the OTR and $q_{{{\text{O}}_{2} }}$ values of these by-product tests, equal to 5.8–6.1 mmol/L/h and 0.7–1.4 mmol/g_CDW_/h, were significantly lower than those obtained in the SM tests under the same aeration conditions (14.1 mmol/L/h and 2.4–5 mmol/g_CDW_/h, respectively), and below the optimal OTR and $q_{{{\text{O}}_{2} }}$ ranges evaluated for the standard medium, probably as a result of the significant presence of suspended solids. Therefore, the lower performances obtained in tests BP-1 and BP-2 could be ascribed not only to the different types of carbohydrates and proteins utilized but also to the lower availability of oxygen.

To better investigate the effect of OTR and $q_{{{\text{O}}_{2} }}$ on the performances of the by-product tests, two further tests (BP-3 and BP-4) were conducted with the molasses-CMBM medium (the most interesting one from an economical point of view), under aeration conditions tentatively designed so as to cover—with the inclusion also of BP-2—OTR and $q_{{{\text{O}}_{2} }}$ ranges (6–13 mmol/L/h and 0.8–3.1 mmol/g_CDW_/h, respectively) close to those identified as optimal for the SM tests (7–15 mmol/L/h and 1.4–7.9 mmol_O2_/g_CDW_/h). In these molasses-CMBM tests, the increase in OTR and $q_{{{\text{O}}_{2} }}$ did not lead to any improvement of the BD production performances: the BD yield varied in the 0.20–0.25 g/g range (45–55% decrease in comparison with the optimal SM tests), the average productivity was equal to 0.42–0.43 g/h/L (50% decrease) and the maximum BD rate was 1.1 g/h/L (27% decrease). These findings suggest that further research is needed to optimize the aeration conditions of the process using these byproducts.

The evaluation of the BD production performances obtained with the molasses-CMBM medium was integrated by a rough estimation of the variation in operating and capital costs associated to the replacement of the standard medium with the molasses-CMBM one (Table 3). The industrial costs for the purchase of the medium components, obtained from various international providers of chemicals, were set to 0.35 $/kg for glucose, 1.5 $/kg for beef extract, 1.2 $/kg for soy peptone, 0.07 $/kg for molasses, 0.25 $/kg for CMBM. The cost of medium preparation per kg of BD produced was evaluated as (total medium cost)/[(sugar concentration in the medium) ∙ (BD/sugar yield)]. The bioreactor size was evaluated as (requested BD production)/(integral BD productivity), taking a 1 t/month target BD production as a reference value. The bioreactor cost was taken equal to 15,000 $/m^3^ (average value of two quotations obtained for this purpose). The plant lifetime was assumed to be equal to 20 years, and the bioreactor cost was uniformly distributed over the 20-year life span. The capital cost per kg of BD produced was evaluated as (yearly capital cost)/(BD mass produced yearly). As shown in Table 3, taking into account the decrease in BD yield and productivity associated to the shift from standard medium to the molasses-CMBM medium, the medium change determined a 50% decrease in medium cost and a 70% increase in capital cost of the bioreactor, with an overall decrease of the cost per kg of BD produced equal to about 45%. This preliminary analysis does not take into account other costs that should not change significantly as a result of the change in medium composition, such as aeration, sterilization and BD separation costs.

**Table 3:** Preliminary evaluation of the change in medium cost and bioreactor capital cost associated to the switch from standard medium to agricultural byproduct-based medium

Parameter	Units	Standard medium tests	Byproduct-based tests
Glucose concentration (kg/m^3^)	kg/m^3^	40	–
Beef extract concentration (kg/m^3^)	kg/m^3^	10	–
Soy peptone concentration (kg/m^3^)	kg/m^3^	10	–
Molasses concentration (kg/m^3^)	kg/m^3^	–	74
CMBM concentration (kg/m^3^)	kg/m^3^	–	30
BD/sugar yield (kg/kg)	kg/kg	0.44	0.235
BD productivity (kg/m^3^/day)	kg/m^3^/day	21.8	13.0
Total medium cost ($/m^3^)	$/m^3^	41	13
Reactor volume (m^3^)	m^3^	1.53	2.57
Medium cost ($/kg_BD_ _produced_)	$/kg_BD_ _produced_	2.33	1.20
Bioreactor capital cost ($/kg_BD_ _produced_)	$/kg_BD_ _produced_	0.10	0.16
Medium + bioreactor cost ($/kg_BD_ _produced_)	$/kg_BD_ _produced_	2.42	1.36

## Conclusions

In this work, the optimization of the aeration conditions relative to a process of BD bioproduction was applied to a non-pathogenic microorganism, *Bacillus licheniformis*. The optimal OTR range, equal to 7–15 mmol/L/h, led to a BD yield equal to 0.44 g/g and productivity of 0.91 g/L/h, using a standard laboratory medium based on glucose, beef extract and peptone. Under the same conditions, the optimal specific O_2_ uptake rate $q_{{{\text{O}}_{2} }}$—a useful scale-up parameter for microaerobic fermentations—varied in the 1.4–7.9 mmol_O2_/g_CDW_/h range, whereas the respiratory quotient RQ—a suitable control parameter—ranged from 1.8 to 2.5. The replacement of the standard medium with a cheap medium based on molasses and chicken meat and bone meal led to a 50% decrease in both BD yield and productivity. A preliminary economic analysis indicated that, taking into account the observed decrease in performances, the use of the agricultural byproduct-based medium can potentially decrease of about 45% the process costs affected by the change in fermentation medium (medium preparation and bioreactor investment cost). Further research is needed to increase the yield and productivity of BD on byproduct-based raw materials.

## Additional file

**Additional file 1: Table S1.** Dynamic method utilized for the experimental evaluation of the mass transfer coefficient *k*_L_*a*. **Table S2**. Evaluation of the volumetric mass transfer coefficient (*k*_L_*a*) by means of empirical correlations. **Table S3.** Evaluation of the optimal agitation/aeration condition using response surface methodology. **Figure S1.** Experimental versus *k*_L_*a*-based *OTR* values. **Figure S2**. 3D plot of overall BD yield versus air flow rate (*Q*_*G*_) and agitation rate (*N*) obtained by response surface methodology.

## Abbreviations

AA: acetic acid
AC: acetoin
BD: 2,3-Butanediol
BP: byproducts
CCCD: Central Composite Circumscribed Design
CDW: cell dry weight
*C*_*G*_: oxygen concentrations in the gas phase
$C_{G}^{IN}$: oxygen concentrations in the entering gas phase
$C_{G}^{OUT}$: oxygen concentrations in the exiting gas phase
*C*_L_: oxygen concentrations in the liquid phase
CMBM: chicken meat and bone meal
DoE: design of experiments
EtOH: ethanol
GLY: glycerine
*k*_L_*a*: volumetric mass transfer coefficient
$m_{{{\text{O}}_{2} }}$: gas/liquid partition coefficient for oxygen
N: impeller rotational speed
NAD^+^: nicotinamide adenine dinucleotide
NADH/NAD^+^: nicotinamide adenine dinucleotide ratio
OD: optical density
OTR: volumetric oxygen transfer rate
OUR: volumetric oxygen uptake rate
*P*_BD_: average BD productivity
*Q*_G_: gas flow rate
$Q_{\text{G}}^{\text{IN}}$: entering gas volumetric flow rate
$Q_{\text{G}}^{\text{OUT}}$: exiting gas volumetric flow rate
$q_{{{\text{O}}_{2} }}$: oxygen-specific uptake rate
*r*_BD_: maximum BD production rate
*r*_GL_: glucose consumption rate
RQ: respiratory quotient, defined as the molar ratio of carbon dioxide production rate to O_2_ uptake rate
RSM: response surface methodology
SM: standard medium
*V*_G_: volume of gas
*V*_L_: volume of liquid
*Y*_AC_: acetoin yield
*Y*_BD_: BD yield
*Y*_GLY_: glycerol yield

## References

[1] Harvey BG, Merriman WW, Quintana RL (2016). Renewable gasoline, solvents, and fuel additives from 2,3-butanediol. Chemsuschem.

[2] Celińska E, Grajek W (2009). Biotechnological production of 2,3-butanediol-current state and prospects. Biotechnol Adv.

[3] Koutinas AA, Vlysidis A, Pleissner D, Kopsahelis N, Lopez Garcia I, Kookos IK (2014). Valorization of industrial waste and by-product streams via fermentation for the production of chemicals and biopolymers. Chem Soc Rev.

[4] Gräfje H, Körnig W, Weitz H-M, Reiß W, Steffan G, Diehl H (2000). Butanediols, butenediol, and butynediol.

[5] Rebecchi S, Zanaroli G, Fava F (2016). 2,3-butanediol production from biowastes with *Bacillus licheniformis*: a preliminary study. Chem Eng Trans.

[6] Ji XJ, Huang H, Ouyang PK (2011). Microbial 2,3-butanediol production: a state-of-the-art review. Biotechnol Adv.

[7] Dürre P, Eikmanns BJ (2015). C1-carbon sources for chemical and fuel production by microbial gas fermentation. Curr Opin Biotechnol.

[8] Tian Y, Fan Y, Liu J, Zhao X, Chen W (2016). Effect of nitrogen, carbon sources and agitation speed on acetoin production of *Bacillus subtilis* SF4-3. Electron J Biotechnol.

[9] Yang T, Rao Z, Zhang X, Lin Q, Xia H, Xu Z (2011). Production of 2,3-butanediol from glucose by GRAS microorganism *Bacillus amyloliquefaciens*. J Basic Microbiol.

[10] Jurchescu IM, Hamann J, Zhou X, Ortmann T, Kuenz A, Prüße U (2013). Enhanced 2,3-butanediol production in fed-batch cultures of free and immobilized *Bacillus licheniformis* DSM 8785. Appl Microbiol Biotechnol.

[11] Clements LD, Miller BS, Streips UN (2002). Comparative growth analysis of the facultative anaerobes *Bacillus subtilis*, *Bacillus licheniformis*, and *Escherichia coli*. Syst Appl Microbiol.

[12] Shariati P, Mitchell WJ, Boyd A, Priest FG (1995). Anaerobic metabolism in *Bacillus licheniformis* NCIB 6346. Microbiology.

[13] Wang Z, Zhuge J, Fang H, Prior BA (2001). Glycerol production by microbial fermentation: a review. Biotechnol Adv.

[14] Biswas R, Yamaoka M, Nakayama H, Kondo T, Yoshida KI, Bisaria VS (2012). Enhanced production of 2,3-butanediol by engineered *Bacillus subtilis*. Appl Microbiol Biotechnol.

[15] Voloch M, Jansen NB, Ladisch MR, Tsao GT, Narayan R, Rodwell VW, Moo-Young M, Blanch HVìW, Drew S, Wang DIC (1985). 2,3-Butanediol. Comprehensive biotechnology. The practice of biotechnology: current commodity products.

[16] Converti A, Perego P, Del Borghi M (2003). Effect of specific oxygen uptake rate on *Enterobacter aerogenes* energetics: carbon and reduction degree balances in batch cultivations. Biotechnol Bioeng.

[17] Garcia-Ochoa F, Gomez E (2009). Bioreactor scale-up and oxygen transfer rate in microbial processes: an overview. Biotechnol Adv.

[18] Ju L, Chase GG (1992). Improved scale-up strategies of bioreactors. Bioprocess Eng.

[19] Byun TG, Zeng AP, Deckwer WD (1994). Reactor comparison and scale-up for the microaerobic production of 2,3-butanediol by *Enterobacter aerogenes* at constant oxygen transfer rate. Bioprocess Eng.

[20] Zeng AP, Byun T, Posten C, Deckwer W (1994). Use of respiratory quotient as a control parameter for optimum oxygen supply production under microaerobic conditions. Biotechnol Bioeng.

[21] Franzén CJ (2003). Metabolic flux analysis of RQ-controlled microaerobic ethanol production by *Saccharomyces cerevisiae*. Yeast.

[22] Zeng A, Biebl H, Deckwer W (1990). 2,3-Butanediol production by *Enterobacter aerogens* in continuous culture; role of oxygen supply. Appl Microbiol Biotechnol.

[23] Ramachandran KB, Hashim MA, Fernandez AA, Lumpur K (1990). Kinetic study of 2,3-butanediol production by *Klebsiella oxytoca*. J Ferment Bioeng.

[24] Silveira MM, Schmidell W, Berbert MA (1993). Effect of the air supply on the production of 2,3-butanediol by *Klebsiella pneumoniae* NRRL-B199. J Biotechnol.

[25] Qureshi N, Cheryan M (1989). Effects of aeration on 2,3-butanediol production from glucose by *Klebsiella oxytoca*. J Ferment Bioeng.

[26] Li L, Zhang L, Li K, Wang Y, Gao C, Han B (2013). A newly isolated *Bacillus licheniformis* strain thermophilically produces 2,3-butanediol, a platform and fuel bio-chemical. Biotechnol Biofuels.

[27] Ripoll V, de Vicente G, Moran B, Rojas A, Segarra S, Montesinos A (2015). Novel biocatalysts for glycerol conversion into 2,3-butanediol. Process Biochem.

[28] Lin CSK, Pfaltzgraff LA, Herrero-Davila L, Mubofu EB, Abderrahim S, Clark JH (2013). Food waste as a valuable resource for the production of chemicals, materials and fuels. Current situation and global perspective. Energy Environ Sci.

[29] Deshmukh AN, Nipanikar-Gokhale P, Jain R (2016). Engineering of *Bacillus subtilis* for the production of 2,3-butanediol from sugarcane molasses. Appl Biochem Biotechnol.

[30] Guragain YN, Chitta D, Karanjikar M, Vadlani PV (2017). Appropriate lignocellulosic biomass processing strategies for efficient 2,3-butanediol production from biomass-derived sugars using *Bacillus licheniformis* DSM 8785. Food Bioprod Process.

[31] Sikora B, Kubik C, Kalinowska H, Gromek E, Białkowska A, Jędrzejczak-Krzepkowska M (2016). Application of byproducts from food processing for production of 2,3-butanediol using *Bacillus amyloliquefaciens* TUL 308. Prep Biochem Biotechnol.

[32] Białkowska AM, Gromek E, Krysiak J, Sikora B, Kalinowska H, Jędrzejczak-Krzepkowska M (2015). Application of enzymatic apple pomace hydrolysate to production of 2,3-butanediol by alkaliphilic *Bacillus licheniformis* NCIMB 8059. J Ind Microbiol Biotechnol.

[33] Kang IY, Park JM, Hong WK, Kim YS, Jung YR, Kim SB (2015). Enhanced production of 2,3-butanediol by a genetically engineered *Bacillus* sp. BRC1 using a hydrolysate of empty palm fruit bunches. Bioprocess Biosyst Eng.

[34] Li L, Li K, Wang K, Chen C, Gao C, Ma C (2014). Efficient production of 2,3-butanediol from corn stover hydrolysate by using a thermophilic *Bacillus licheniformis* strain. Bioresour Technol.

[35] Yang T, Rao Z, Zhang X, Xu M, Xu Z (2015). Economic conversion of spirit-based distillers’ grain to 2,3-butanediol by *Bacillus amyloliquefaciens*. Process Biochem.

[36] Mekonnen T, Mussone P, Bressler D (2014). Valorization of rendering industry wastes and co-products for industrial chemicals, materials and energy: review. Crit Rev Biotechnol.

[37] Cascarosa E, Gea G, Arauzo J (2012). Thermochemical processing of meat and bone meal: a review. Renew Sustain Energy Rev.

[38] Liang Y, Garcia RA, Piazza GJ, Wen Z (2011). Nonfeed application of rendered animal proteins for microbial production of eicosapentaenoic acid by the fungus *Pythium irregulare*. J Agric Food Chem.

[39] Pleissner D, Venus J (2016). Utilization of protein-rich residues in biotechnological processes. Appl Microbiol Biotechnol.

[40] Solaiman D, Garcia RA, Ashby R, Piazza GJ, Steinbüchel A (2011). Rendered-protein hydrolysates for microbial synthesis of cyanophycin biopolymer. N Biotechnol..

[41] Scargiali F, Busciglio A, Grisafi F, Brucato A (2010). Simplified dynamic pressure method for *k*_L_*a* measurement in aerated bioreactors. Biochem Eng J.

[42] Pinelli D, Liu Z, Magelli F (2010). Analysis of *k*_L_*a* measurement methods in stirred vessels: the role of experimental techniques and fluid dynamic models. Int J Chem React Eng..

[43] Nilegaonkar S, Bhosale SS, Kshirsagar DDD, Kapadi AH (1992). Production of 2,3-butanediol from glucose by *Bacillus licheniformis*. World J Microbiol Biotechnol.

[44] Bradford MM (1976). A rapid and sensitive method for the for the quantitation of microgram quantities of protein utilizing the principle of protein dye-binding. Anal Biochem.

[45] Van’t Riet K (1979). Review of measuring methods and results in nonviscous gas-liquid mass transfer in stirred vessels. Ind Eng Chem Process Des Dev..

[46] Paul EL, Atiemo-obeng VA, Kresta SM (2004). Handbook of industrial mixing: science and practice.

[47] Dai JY, Zhao P, Cheng XL, Xiu ZL (2015). Enhanced production of 2,3-butanediol from sugarcane molasses. Appl Biochem Biotechnol.

[48] Yang T, Zhang X, Rao Z, Gu S, Xia H, Xu Z (2012). Optimization and scale-up of 2,3-butanediol production by *Bacillus amyloliquefaciens* B10-127. World J Microbiol Biotechnol.

[49] Lee SM, Oh BR, Park JM, Yu A, Heo SY, Hong WK (2013). Optimized production of 2,3-butanediol by a lactate dehydrogenase-deficient mutant of *Klebsiella pneumoniae*. Biotechnol Bioprocess Eng.

[50] Xin F, Basu A, Weng MC, Yang KL, He J (2016). Production of 2,3-butanediol from sucrose by a *Klebsiella species*. Bioenergy Res..

[51] Derringer G, Suich R (1980). Simultaneous optimization of several response variables. J Qual Technol..

[52] Ge Y, Li K, Li L, Gao C, Zhang L, Ma C (2016). Contracted but effective: production of enantiopure 2,3-butanediol by thermophilic and GRAS *Bacillus licheniformis*. Green Chem. Royal Society of Chemistry.

[53] Silveira MM, Schmidell W, Berbert MA (1993). Effect of the air supply on the production of 2,3-butanediol by *Klebsiella pneumoniae* NRRL B199. J Biotechnol.

[54] Anvari M, Mohammad R, Motlagh S (2011). Enhancement of 2,3-butanediol production by *Klebsiella oxytoca* PTCC 1402. J Biomed Biotechnol..

[55] Hazeena SH, Pandey A, Binod P (2016). Evaluation of oil palm front hydrolysate as a novel substrate for 2,3-butanediol production using a novel isolate *Enterobacter cloacae* SG1. Renew Energy..

[56] Wang Q, Chen T, Zhao X, Chamu J (2012). Metabolic engineering of thermophilic *Bacillus licheniformis* for chiral pure d-2,3-butanediol production. Biotechnol Bioeng.

[57] Zeng AP, Biebl H, Deckwer WD (1991). Production of 2,3-butanediol in a membrane bioreactor with cell recycle. Appl Microbiol Biotechnol.

[58] Ma C, Wang A, Qin J, Li L, Ai X, Jiang T (2009). Enhanced 2,3-butanediol production by *Klebsiella pneumoniae* SDM. Appl Microbiol Biotechnol.

[59] Jung MY, Ng CY, Song H, Lee J, Oh MK (2012). Deletion of lactate dehydrogenase in *Enterobacter aerogenes* to enhance 2,3-butanediol production. Appl Microbiol Biotechnol.

[60] Ji XJ, Huang H, Zhu JG, Ren LJ, Nie ZK, Du J (2010). Engineering *Klebsiella oxytoca* for efficient 2,3-butanediol production through insertional inactivation of acetaldehyde dehydrogenase gene. Appl Microbiol Biotechnol.

