# Supplementary material for: Effect of oxygen mass transfer rate on the production of 2,3-butanediol from glucose and agro-industrial byproducts by Bacillus licheniformis ATCC9789
Source: Biotechnol Biofuels. 2018 May 23;11:145. doi: 10.1186/s13068-018-1138-4 (PMC5964669; doi:10.1186/s13068-018-1138-4)
Supplement: Supplementary file 1 — Additional file 1: Table S1. Dynamic method utilized for the experimental evaluation of the mass transfer coefficient kLa. Table S2. Evaluation of the volumetric mass transfer coefficient (kLa) by means of empirical correlations. Table S3. Evaluation of the optimal agitation/aeration condition using response surface methodology. Figure S1. Experimental versus kLa-based OTR values. Figure S2. 3D plot of overall BD yield versus air flow rate (QG) and agitation rate (N) obtained by response surface methodology. [file 13068_2018_1138_MOESM1_ESM.docx]

**Effect of oxygen mass transfer rate on the production of 2,3-butanediol from glucose and agro-industrial byproducts by *Bacillus licheniformis* ATCC9789**

Stefano Rebecchi, Davide Pinelli, Giulio Zanaroli, Fabio Fava, Dario Frascari

**ADDITIONAL FILE 1**

**Table S1 Dynamic method utilized for the experimental evaluation of the mass transfer coefficient *k_L_a***

| Different dynamic and steady-state methods can be used to experimentally determine *k_L_a*. In this study, *k_L_a* was measured in the actual fermentation medium, before each fermentation test, for each condition studied in the BD bioproduction experiments, using a variation of the conventional dynamic method. This method is based on the measurement of the transient dissolved oxygen concentration in the medium in absence of biomass (no oxygen consumption) while applying a step change between two different conditions (from zero oxygen in the liquid to air saturation or vice versa). For all experimental conditions, *k_L_a* was measured by stripping oxygen from the fermentation medium through nitrogen sparging, then air was fed at the flow rate of the studied agitation/aeration condition. The step change from air saturated liquid to oxygen absence by N_2_ sparging at the same Q_G_ was also performed in some cases. As very similar *k_L_a* values were obtained with the two approaches, the two estimates were averaged. The experimental *k_L_a* evaluation method is based on the O_2_ mass balance in the liquid written for a perfectly mixed system in semi-batch mode with no oxygen consumption:  $\frac{dC_{L}}{dt}= k_{L}a \cdot( \frac{C_{G}}{m}-C_{L})$ (S1)  Integrating Eq. (S1) between two different times, the following equation is obtained:  $\ln\frac{\left( {C_{G2}}/m \right)-C_{L2}}{\left( {C_{G1}}/m \right)-C_{L1}}=-k_{L}a\cdot(t_{2}-t_{1})$ (S2)  Therefore, *k_L_a* can be assessed as the slope of a graph of the logarithm of the concentrations versus time. However, if the input gas is not a single component but a mixture of oxygen and nitrogen, characterized by different solubilities and diffusivities, the slope obtained may differ from the actual *k_L_a*. The influence of this aspect on the estimated slope can be analytically predicted. On the other hand, if the mass transfer is not too high (*k_L_a* < 540 1/h), the deviation between the real and experimental *k_L_a* is generally < 2% [42]. A more severe limitation in all dynamic methods is the probe time lag or electrode response time, that influences the instantaneous slope of the curve. This effect can be neglected if the probe time lag is one order of magnitude lower than the mass transfer characteristic time (1/*k_L_a*). This constraint is easily satisfied in case of relatively low *k_L_a* values, such as those in this work where fully aerobic conditions must be avoided. In this work the probe response time was equal to 10 s, corresponding to a *k_L_a* threshold value of 0.01 1/s (36 1/h) for complete negligibility of the probe response time. On the other hand, Scargiali [41] noticed that if probe time lag is not fully negligible, an initial deviation from the straight line was observed (lower slope), but the correct value could still be obtained by discharging the initial non-linear data and fitting only those corresponding to the linear portion of the curve. In this condition, the maximum *k_L_a* that can be assessed with an acceptable accuracy can be calculated as 0.6/τ_probe_, corresponding to a *k_L_a* of 0.06 1/s (216 1/h). In this work, in all the tested conditions the *k_L_a* values resulted lower than the limit of acceptable accuracy suggested by Scargiali [41] (216 1/h) whereas – among the micro-aerobic tests leading to satisfactory BD production performances - only two studied led to calculated *k_L_a* values slightly higher than the limited for complete negligibility (36 1/h). |
| --- |

**Table S2 Evaluation of the volumetric mass transfer coefficient (*k_L_a*) by means of empirical correlations**

| The theoretical evaluation of *k_L_a* was based on the most common correlation utilized in the literature to estimate *k_L_a* in a stirred tank reactor [46], which relates *k_L_a* to the mechanical power dissipated for aeration (*P_g_*) and to the inlet gas flow rate expressed in terms of superficial velocity (*v_s_*), defined as ratio of the gas flow rate to the tank transversal section:  $k_{L}a=K\cdot\left( \frac{P_{g}}{V} \right)^{\alpha}\cdot v_{s}^{\beta}$ (S3)  where *K*, *α*, *β* are empirical coefficients that depend on liquid properties, bioreactor geometry and impeller type. The gassed power input *P_g_*, in turn, can be estimated according to the following equation:  $P_{g}=RPD\cdot N_{p}\cdot N^{3}\cdot D^{5}\cdot\rho_{l}$ (S4)  where *N_p_* is the impeller power number (about 5 for the six-blade Rushton turbine used in this study, in case of turbulent regime), *D* the impeller diameter, $\rho_{l}$ the liquid density and *RPD* the relative power demand. The latter and can be calculated as $RPD=0.18\cdot{Fr}^{-0.25}\cdot{Fl}^{-0.2}$, where $Fr=N^{2}\cdot D/g$ is the Froude number and $Fl=Q_{G}/(ND^{3})$ is the gas flow number. Therefore, once the tank and impeller geometry are fixed, *k_L_a* is an increasing function of the operational conditions *N* and *Q_G_* (*k_L_a* = *const* ∙ *N ^2.7α^ ∙ Q_G_ ^β−0.2α^*). The *k_L_a* values in the experimental conditions tested in this work were predicted using two sets of *K, α* and *β*: the first one (K = 0.026, α = 0.40, β = 0.50; [45]) has been largely used in the literature for a standard bioreactor, whereas the second was more recently proposed by Scargiali (K = 0.0037, α = 0.585, β = 0.35; [41]). Density and viscosity were approximated to those of pure water at 30°C.  The fermenter used for this study was provided with four 1-cm baffles on the bioreactor walls. Agitation was transmitted to the liquid by a single six-blade Rushton turbine located at a 0.035 m distance from the vessel bottom, and characterized by a 0.050 m diameter (D), a 0.010 m blade height and a 0.015 m blade width. |
| --- |

**Fig. S1 –** Experimental versus *k_L_a*-based *OTR* values

**Table S3 Evaluation of the optimal agitation/aeration condition using Response Surface Methodology**

| The evaluation of the optimal OTR was integrated by an assessment of the optimal operating condition in terms of impeller rotational speed *N* and air flow rate *Q_G_*, based on Response Surface Methodology (RSM). The RSM and the statistical analysis, carried out with MODDE 10.1 software (Umetrics, Umeå, Sweden), were based on the data relative to the CCCD tests (SM-2 to SM-10, Table 1), and were applied to the overall BD yield (*Y_BD_*) and average productivity (*P_BD_*), considered the two most relevant performance parameters. For both *Y_BD_* and *P_BD_* a best-fitting second-degree polynomial equation of the operating conditions *N* and *Q_G_* was calculated:  *Y_BD_* = 0.1017 + 0.2273∙*Q_G_* + 0.0018∙*N* - 0.2706∙*Q_G_*^2^ + 0.0000∙*N*^2^ - 0.0004∙*Q_G_∙N* (S5)  *P_BD_* = -0.4504 + 0.9522∙*Q_G_* + 0.0053∙*N* - 0.2429∙*Q_G_*^2^ + 0.0000∙*N*^2^ - 0.0019∙*Q_G_∙N* (S6)  The analysis of variance (ANOVA) indicated that both polynomial equations were in good agreement with the experimental results (data not shown). As an example, the 3D plot relative to the overall BD yield (Eq. S5) is shown in Fig. S2.  In order to identify the optimal (*N*, *Q_G_*) combination, a combined yield-productivity surface was evaluated according to the desirability approach for multiple-response problems [60]. The maximum of the combined surface was obtained for *N*= 462 rpm and *Q_G_*= 0.1 L/min. The optimized *k_L_a* corresponding to this aeration condition was evaluated by means of Eqs. (S3) and (S4), using the following set of constants *K, α* and *β*, obtained by best-fit of the experimental *k_L_a* values reported in Table 1: *K*  = 0.000581, *α* = 1.30, *β* = 0.609. The optimal *k_L_a* was equal to  33.8 1/h. The corresponding *k_L_a*-based *OTR* was estimated according to Eq. (4). Lastly, the actual *OTR* - evaluated from the *k_L_a*-based *OTR* on the basis of the correlation reported in Fig. S1 – was equal to 8.5 mmolO_2_/L/h. This result is in good agreement with the optimal *OTR* range obtained from the CCCD tests, equal to 7-11 mmolO_2_/L/h. |
| --- |

**Fig. S2 –** 3D plot of overall BD yield versus air flow rate (*Q_G_*) and agitation rate (*N*) obtained by Response Surface Methodology.
